# Orchestrating gameplay in Dutch physical education: how and why teachers regulate task difficulty

**DOI:** 10.3389/fspor.2026.1837783

**Published:** 2026-05-29

**Authors:** Joyce E. Burger, Marjan Kok, Jeroen B. J. Smeets, Katinka van der Kooij

**Affiliations:** Department of Human Movement Sciences, Faculty of Behavioural and Movement Sciences, Vrije Universiteit Amsterdam, Amsterdam Movement Sciences, Amsterdam, Netherlands

**Keywords:** differentiation, motivation, physical education, physical literacy, task difficulty

## Abstract

**Introduction:**

Physical education (PE) teachers play a central role in shaping students' physical literacy, including motivation, confidence, physical competence, knowledge, and understanding that supports lifelong physical activity. Differentiation of task difficulty during lessons is widely considered essential in this process. Yet in game-based lessons, what challenges one student may overwhelm or bore another. To better understand how and why teachers differentiate task difficulty, this qualitative study explored how junior secondary school PE teachers adapt task difficulty during game-based lessons.

**Methodology:**

Fourteen teachers participated in stimulated recall interviews based on 20-minute GoPro recordings of their own lessons. We analyzed the data using reflexive thematic analysis.

**Results:**

Teachers described differentiation as an ongoing, situated process of monitoring and shaping students' engagement by verbal guidance, rather than primarily modifying task constraints (e.g., rules or equipment). Teachers emphasized fostering inclusive participation, cultivating a positive pedagogical climate, using praise to reinforce desired behaviors, and regulating gameplay by stepping in and out.

**Discussion:**

Differentiation most often occurred through verbal feedback, with particular attention to supporting students perceived as less confident, while engaging higher-skilled students in role-model or leadership roles. In their reasoning, teachers placed students' confidence and motivation at the forefront, describing them as important building blocks from which physical competence may develop. We describe PE teachers as “motivational coaches”, as their decision-making prioritizes students' emotional and relational experiences, with the expectation that skill development will follow. This contrasts with earlier research emphasizing skill-focused teaching. However, it can be questioned whether optimal motor learning occurs if teachers reduce task difficulty to support confidence. We suggest that acknowledging students' effort and progress while challenging a student may support both motivation and learning. Since challenge is a subjective concept, it is important to foreground students' experiences of challenge in game-based PE in future research.

## 1 Introduction

Physical education (PE) teachers are responsible for designing lessons that meet individual students’ needs and foster each student's potential (1). This aim aligns with teachers striving for enabling students to realize their movement potential and enhancing their quality of life, equal to the idea of developing physical literacy (2). Physical literacy is defined as the motivation, confidence, physical competence, knowledge, and understanding to maintain lifelong physical activity. A key aspect of meeting individual students’ needs is for PE teachers to adjust task difficulty to match each student's skill level (i.e., differentiation) (3). When tasks are too easy or too difficult, this can lead to disengagement, and students may fail to reach their full learning potential (4). Game-based lessons, which form a major component of PE curricula worldwide (5), pose particular challenges for differentiation of task difficulty because teachers must manage diverse skill levels within dynamic, interactive team settings (6).

The teaching games for understanding framework offers practical strategies to differentiate task difficulty by manipulating game complexity, while preserving meaningful tactical learning situations (7). Within this framework, teachers are expected to act as designers of the learning environment, constructing small-sided games by manipulating task constraints such as offense-defense ratio, rules, equipment, and court sizes (8, 9). During gameplay, teachers are supposed to adopt a facilitative role by monitoring gameplay dynamics and adjusting task difficulty through questioning or by modifying task constraints in response to students’ tactical decisions, thereby promoting meaningful student–environment interactions while maintaining a more hands-off approach (9, 10).

Although teaching games for understanding proposes practical ways to manipulate task constraints during gameplay, differentiating task difficulty in dynamic game-based environments remains a complex undertaking (6). A task that is appropriately challenging for one student (e.g., an attacker) may be too easy or too difficult for another (e.g., a defender). This requires teachers to act on their in-the-moment estimates of how individual students will respond and how this will affect others and game dynamics. Two theoretical perspectives explain why such real-time differentiation of task difficulty matters to the student's learning and motivation. Vygotsky's zone of proximal development highlights that learning is optimized when tasks are set just beyond a student's current skill level and supported through guided interaction (11). Self-determination theory emphasizes that fulfillment of the students’ psychological need for competence depends on an appropriate match between task demands and skill level (12). Fulfillment of this need for competence results in motivated task engagement. However, the relationship between motivation and learning remains unresolved. Does task difficulty within the zone of proximal development foster motivation by satisfying the need for competence? Or must this need first be met before high task difficulty can support learning? Consequently, it is unclear whether teachers can foster learning and motivation simultaneously or whether, in practice, they must prioritize one over the other.

Although task difficulty differentiation is woven into the design of teaching games for understanding interventions, existing studies have focused on comparing these interventions to technical or traditional teaching approaches across a range of outcomes, including: gameplay opportunities (13), game performance (14–16), student motivation (14, 16), enjoyment (15), and perceived competence (15). For instance, Estriga et al. (13) designed a teaching games for understanding handball unit using task constraint modifications such as a softer ball and adjusted scoring rules. They found that reducing task difficulty by adding an extra player to create a numerical advantage increased both individual attacking actions (more passing, catching, and goal scoring opportunities) and relational actions (more open passing lanes) compared to equal-numbered play. Alcalá and Garijo (14) compared a teaching games for understanding approach to teaching basketball, floorball, and handball with a technical approach. They found higher experiences of motivation in the teaching games for understanding group, where task difficulty was differentiated through modifications such as adapting game structure to student needs, manipulating tactical complexity, and creating situations of offensive and defensive superiority. Morales-Belando and Arias-Estero (15) assessed students before and after a teaching games for understanding floorball unit and found improvements in both learning and motivation. Within this intervention, task difficulty was differentiated primarily through varying the number of pupils, practice area, and gameplay rules to integrate technical and tactical elements of gameplay in a stepwise manner. Most recently, a study compared a teaching games for understanding based football group to a traditional skill-drill control group according to a pre- and post-test design (16). They found that the teaching games for understanding group scored higher on decision-making, skill execution, and off-the-ball movement at post-test, as well as on intrinsic motivation. Together, these findings suggest that differentiation of task difficulty benefits both learning and motivation. However, how teachers differentiate task difficulty in the moment during game-based lessons, and how learning and motivation interact in those moments, remains unclear.

While the design of task difficulty at the lesson level may simultaneously benefit motivation and learning, these goals may conflict in teachers’ moment-to-moment decisions about task difficulty. For instance, a task that is easy enough to keep a struggling student engaged may not provide sufficient challenge for skill development. Regarding why task difficulty is differentiated, it remains unclear which considerations drive these decisions and how teachers weigh students’ tactical responses, motivational states, and skill levels in the moment. Yet understanding these decisions would shed light on how motivation and learning are intertwined in practice. The limited qualitative literature that does address teacher considerations regarding differentiating task difficulty focuses either on a different context, such as swimming lessons (17), or on the PE lesson broadly, revealing that teachers seem to prioritize ‘fun’ to encourage out-of-school physical activity and deliberately limit error feedback to avoid undermining student confidence (18–20).

Thus, studying teachers’ moment-to-moment decisions during game-based lessons is interesting because it provides insight into how teachers target motivation and learning and their interplay. Research on teacher cognition in PE has established that teachers make interactive decisions continuously during instruction, with the greatest proportion of thoughts concerning students’ behaviors, affect, and engagement (21). Elementary school PE teachers teaching for more than five years make instructional decisions with a focus on skill acquisition (22). These decisions are made on impulse, without time to reflect or consult new information, requiring teachers to improvise responses to situations as they unfold (21, 22). To our knowledge, however, no prior study has examined how PE teachers differentiate task difficulty during game-based lessons in naturalistic settings, nor what considerations underpin these decisions. Yet insight into task difficulty differentiation during in-the-moment game-based lessons and the reasoning behind it is a necessary step between teaching games for understanding, offering a practical toolbox, and the intervention studies that drew inspiration from it. Without understanding how task difficulty differentiation is applied in practice and why teachers make these decisions, an important intermediate step is missing. This step involves examining its implementation in game-based practice and the underlying reasoning before designing interventions and comparing them with standard teaching approaches in terms of motivation and learning. Our qualitative study, therefore, differs from previous work by using stimulated recall interviews to capture teachers’ reflections on specific teaching moments and articulate the reasoning behind their actions (23). Rather than focusing on teachers’ general opinions about differentiation across the lesson as a whole, we examine the internal thought processes that shape moment-to-moment decision making during game-based lessons. Our study addresses the following research question: *How and why do teachers adapt task difficulty during junior secondary school game-based physical education lessons?*

## 2 Methodology

### 2.1 Research paradigm

This study is grounded in interpretivism, recognizing multiple, context-dependent realities shaped by teachers’ experiences and values (24). Ontologically, interpretivism adopts a relativist stance, acknowledging reality as socially constructed and plural. Epistemologically, the study aligns with social constructivism, which views knowledge as subjective and co-constructed through interactions with the environment (25). Teachers’ perspectives of ‘task difficulty’ emerge through engagement with PE teacher educators, students, colleagues, and institutional norms.

### 2.2 Participants

Using purposive sampling, 39 PE teachers were recruited to ensure geographical diversity across the Netherlands and representation from various Physical Education Teacher Education (PETE) programs. Fourteen teachers participated in interviews (Table 1); 25 declined due to time constraints, reluctance to appear on video, departmental changes, not teaching, lack of interest, or no reason provided. Recruitment occurred during the Dutch PETE network day, professional connections, researchers’ informal networks, LinkedIn posts, and snowball sampling. Sample adequacy was determined in terms of information power rather than data saturation (26). This approach suggests that smaller sample sizes are sufficient when several conditions of information power are met. These included: a small and specific study aim (understanding differentiation of task difficulty); a homogenous participant population (all participants were PE teachers with ≥ 5 years of secondary school teaching experience, teaching a junior secondary school class); theoretical grounding in established frameworks (teaching games for understanding, self-determination theory, and zone of proximal development); high dialogue quality (stimulated recall interviews based on participants’ own taught lessons and clear instructions); and case-specific rather than comparative analysis. Given these five factors, 14 interviews provided sufficient information power to address the research question.

**Table 1:** Demographics of participating teachers.

Variable	*n* (%) or M (SD)
Age	46 (8) years
Teaching Experience	20 (8) years
Gender	
Female	5 (35%)
Male	9 (64%)
Year Taught	
First	8 (57%)
Second	6 (43%)
Level Taught	
Pre-vocational secondary education	4 (29%)
Senior general secondary education	2 (14%)
Pre-university education	4 (29%)
Pre-vocational secondary education/senior general secondary education/pre-university education	2 (14%)
Pre-vocational secondary education/senior general secondary education	1 (7%)
Senior general secondary education/pre-university education	1 (7%)

Categorical variables are presented as numbers (n) and percentages. Continuous variables are presented as means (M) and standard deviations (SD). Age and teaching experience are measured in years at the time of study. Level taught indicates the different levels of junior secondary school education.

Eligibility criteria included: completion of a PETE program at a Dutch University of Applied Sciences, ≥ 5 years’ secondary school teaching experience (excluding internships), and teaching a junior secondary school class. Teachers received detailed information via email on the study aim, filming, consent, and data privacy. No distinction was made between public and private schools, as all Dutch schools are government-funded.

### 2.3 Materials and instruments

Teachers wore a head-mounted GoPro (GoPro HERO 11) during the PE lesson, with a secondary Canon HF R86 camera serving as backup. Footage was transferred to an encrypted university laptop for interviews.

**Figure 1:**
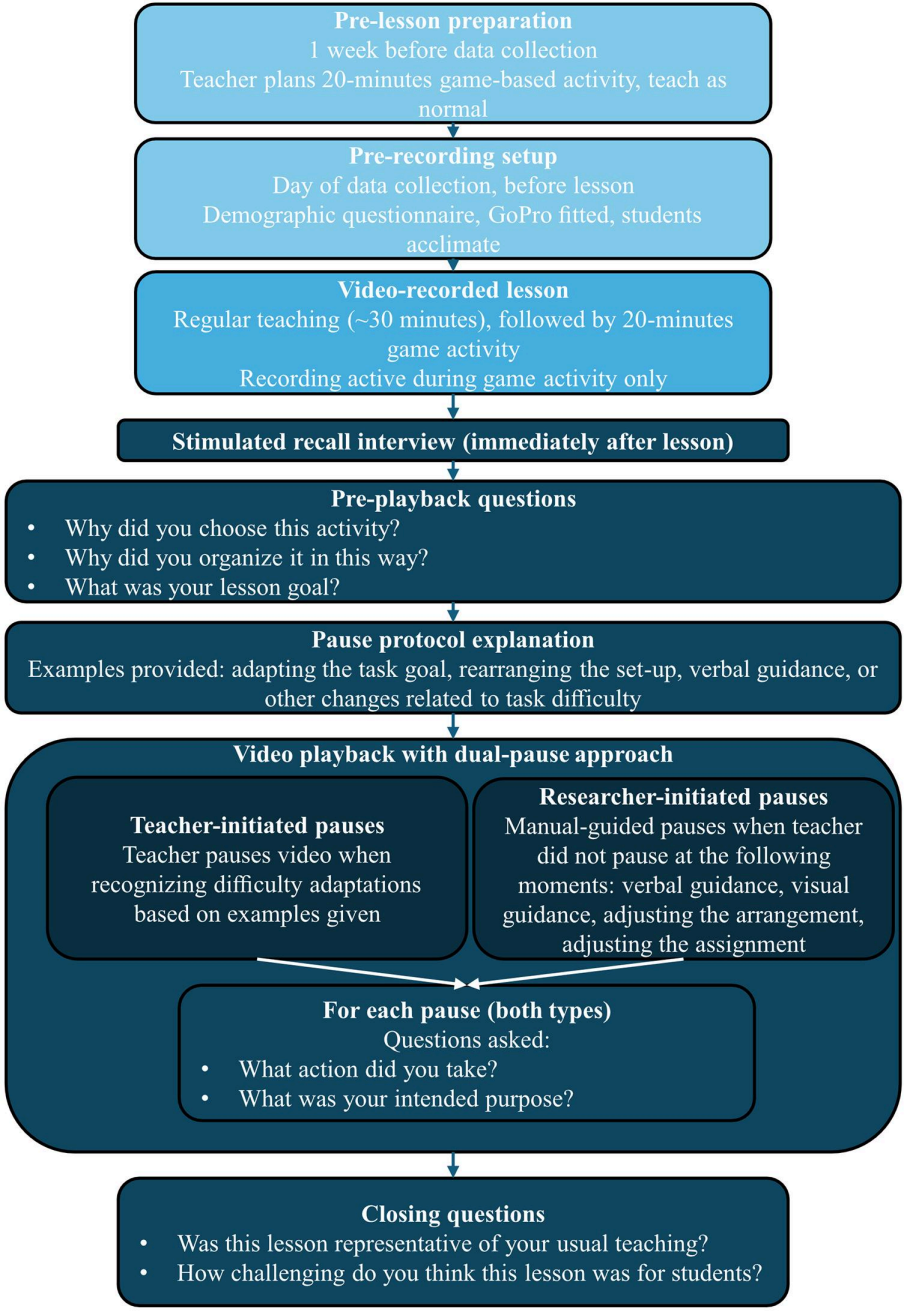
Procedure.

The interview procedure and questions were co-developed by the first and second authors together with an experienced secondary school PE teacher (Figure 1). In this process, common task constraints manipulated during PE lessons were identified and incorporated as examples, and shared with teachers before the interview to support reflection on task difficulty differentiation. Pilot interviews refined the interview procedure and questions. Moreover, the pilot interviews were also used to develop a manual to guide the first author on when to pause the video recording if the teacher did not do so (Table 2). Within this manual, different teacher actions—such as giving verbal guidance, providing visual guidance, adjusting the arrangement, and adjusting the assignment—were defined as categories at which the researcher could pause the video recording if the teacher did not do so. Each category was accompanied by explanations and examples derived from pilot interviews. Pilot data were excluded from analysis.

**Table 2:** Video stop criteria for the first author in case the teacher did not pause the video recording.

Category	Description	Example(s)/notes
Verbal guidance	The teacher responds to a student's comment or question about the game activity or asks questions to assess the student's understanding.	Includes clarifying game rules, questioning students about technical or tactical elements of gameplay, or addressing students' comments that indicate that something is not working. These explanations can be given to a group or an individual student. Off-task remarks such as “I don't feel like it” or “I need to drink water” are not included.
	The teacher makes comments during a game situation, often relating to events on the playing field.	Comments may be positive, highlighting something that is going or has gone well, or serve as feedback.
	The teacher gives a technical or tactical tip or compliment to a team or individual student.	
	The teacher positions students within the game situation.	Directs students to the correct position on the field and may advise them on what to do and what role they can play from that position.
Visual guidance	The teacher visually demonstrates part of the activity.	The teacher may act as a member of a team to model a specific strategy, technique, or gameplay role, applying theory directly in practice.
Adjusting the arrangement	The teacher changes the team composition or the matchup between teams.	May involve moving certain students to a different team, changing who plays against whom, or assigning a different role to a student within their current team.
	The teacher changes the materials used in the lesson.	May involve replacing or modifying sticks, balls, mats, or the size of the playing field.
Adjusting the assignment	The teacher modifies the rules of the game.	Rules may be adjusted in various ways, for example: allowing the ball to bounce once before being returned, or permitting a player to catch the ball before passing it on.
	The teacher changes the objective of the game.	For example, shifting the goal from winning or achieving the highest score to keeping the ball in play as long as possible, creating a rally. Or ensuring that every student has an active role, thereby promoting cooperation.

### 2.4 Procedure

#### 2.4.1 Pre-lesson preparation

One week before data collection, teachers were instructed to dedicate 20 minutes of their PE lesson to a game-based activity (e.g., soccer, basketball, volleyball). They were free to choose which game they picked and how they arranged this game, with only one restriction, namely that they should teach this activity as they normally would.

#### 2.4.2 Video-recorded lesson

Before the start of the video-recorded lesson, the teacher filled out the demographic questionnaire, and the GoPro camera was fitted to the teacher's head. This allowed students to already get used to the GoPro camera without filming taking place.

When the lesson started, the teacher taught their lesson as they normally would, whereby approximately after half an hour, the chosen 20-minute game-based activity was initiated, and video recording was started.

Video recording was stopped after 20 minutes, and the footage was transferred to an encrypted university laptop so that the interview could be conducted immediately after the lesson had finished.

#### 2.4.3 Stimulated recall interview procedure

Interviews were conducted in a quiet room immediately after the teacher had finished their lesson.

Stimulated recall interviews explored teachers’ in-the-moment decisions (23) based on the Go-Pro videos made of their own taught 20-minute game-based PE activity. Before video playback, teachers answered three opening questions: ‘Why did you choose this activity?’, ‘Why did you organize it this way?’, and ‘What was your lesson goal?’ Before video playback, the researcher explained the pausing protocol, asking teachers to pause the recording whenever they made changes that they thought made the activity easier or more difficult. Three examples based on task constraints as mentioned in the teaching games for understanding framework were provided (e.g., adapting the task goal, rearranging the setup (e.g., adapting materials or game rules), and providing verbal guidance) (9), though teachers were also encouraged to pause for any other changes related to task difficulty. For each paused moment, teachers were asked to describe: the action they took, and its intended purpose.

Aside from the teachers being able to pause the recording, the researcher was able to pause the video recording. The manual served as a guideline for the researcher to determine when to pause the video recording when a teacher performed one of these actions, while the teachers themselves did not pause the recording (see Table 2 for a depiction of the stop categories, explanations, and examples). When the researcher paused the video, teachers were asked the same questions as for self-initiated pauses: ‘What action did you take?’ and ‘What was your intended purpose?’ This dual-pause approach ensured comprehensive coverage of task difficulty adaptations while preserving teachers’ own interpretations of these moments.

Interviews concluded with: ‘Was this lesson representative of your usual teaching?’ and ‘How challenging do you think this lesson was for your students?’ See Figure 1 for an overview of the procedure, including the interview questions used in each phase.

All interviews with the teacher were solely performed by the first author and audio recorded, lasting 50–82 min (M = 63).

### 2.5 Data analysis

Clean verbatim transcripts made using Amberscript were verified and analysed with ATLAS.ti (version 24.1.1.30813) using reflexive thematic analysis in an inductive manner (27). Analysis was iterative, involving three stages: (a) familiarization and initial coding, (b) theme generation and code refinement, and (c) reviewing and refining themes. It is important to note that, due to the inductive nature of the reflexive thematic analysis, themes and subthemes did not represent categorizations of teachers’ specific actions, but captured meanings of teachers’ conceptualizations of task difficulty.

#### 2.5.1 Familiarization and initial coding

Transcripts were reread and initially coded, generating descriptive codes for meaning segments. The second and fourth authors engaged with segments of the first transcript to provide reflexive dialogue, helping the first author develop her analytic lens and coding approach. After this initial reflexive discussion, the first author coded the remaining transcripts. Similar codes were merged, recurring codes annotated with memos. As analysis progressed and familiarization with the dataset deepened and coding experience grew, coding evolved from the tenth interview onwards from primarily descriptive surface-level meanings (i.e., ‘changing team composition’) to include interpretation of underlying conceptual meanings (i.e., ‘teacher as guard of equal participation’).

#### 2.5.2 Theme generation and refinement of codes

Initial themes and subthemes captured the meanings of teachers’ conceptualizations of task difficulty. The first nine transcripts were revisited to ensure coding depth and consistency with the evolved analytic approach. Analytical summaries guided this second round of coding. A codebook organized evolving codes and theme definitions, with codes considered complete when labels sufficiently summarized dataset meanings (28) in (27).

#### 2.5.3 Reviewing and refining themes

Themes and subthemes were refined to capture conceptual scope. For example, praise shifted from subtheme to theme due to prominence. Names of (sub)themes were refined for accurate representation of the dataset. A reflexive journal documented analytic decisions and reflections.

#### 2.5.4 Analytical rigor and trustworthiness

Analytical rigor was established through prolonged engagement with the data, including iterative coding and ongoing refinement of themes and subthemes; maintaining an audit trail of analytic decisions through memos; and critical reflection on positionality via a reflexive journal. The first author's sustained immersion in the dataset supported coherence across interviews, while regular reflexive dialogue with co-authors and a PhD peer helped to challenge interpretations and avoid interpretive insularity. Transparent reporting of the analytic process, including examples of coding and theme development, further supports the trustworthiness of the analysis. Consistent with our interpretivist stance, which recognizes multiple, situated readings of the data rather than a single verifiable interpretation, participants did not review transcripts or findings. Within reflexive thematic analysis, the researcher is positioned as the interpretive instrument, and member checking is not treated as a validity requirement (27).

### 2.6 Researchers’ positionality

Reflexive thematic analysis recognizes researcher subjectivity as a resource, and the research team’s diverse backgrounds, informed, rather than undermined, analytic insight.

I, the first author, am a PhD candidate in Human Movement Sciences with a background in quantitative research. To develop qualitative expertise, I completed formal training in qualitative methodology during my doctoral program. Initially, I approached reflexive thematic analysis through a quantitative lens, emphasizing reliability metrics. Through deeper engagement with Braun and Clarke (27) I learned to prioritize subjectivity and meaning-making over seeking agreement.

The second and fourth authors, both with quantitative backgrounds, acted as critical friends throughout the analysis. Their questions often revealed when I reverted to a ‘quantitative headspace’ and helped me realign with qualitative principles. Their contributions were complementary, combining expertise in PETE and psychology. A third critical friend, a fellow PhD candidate, offered additional interpretive dialogue based on related qualitative work in elementary school PE.

Most teachers were previously unknown to me, though two were somewhat familiar (a distant relative and a former colleague of my father). Informal pre-filming conversations often were about shared experiences in sport. As a former high-level athlete, this common ground appeared to foster openness and comfort during interviews, likely contributing to rich, layered data. This openness was further supported by teachers perceiving me as a student conducting a research project. Shared backgrounds and familiarity may also have encouraged participants to assume mutual understanding, leaving certain aspects implicit or presenting practices in a more favorable light.

Finally, as a woman highly engaged in PE during my schooling, I was attentive to participants’ reflections on girls’ participation, which could have shaped my interpretations of the data.

I reflected upon my relationship with participating teachers and their potential consequences, as well as my position as a woman, and how this may have shaped data interpretation during ongoing reflexive journaling throughout data collection and analysis.

### 2.7 Ethical considerations

The study was approved by the local ethical committee (VCWE-2024-103). All teachers provided digital informed consent, following approval from their school boards. Parents/caregivers received information and privacy statements two weeks before student participation. Students whose parents or who themselves wished to opt out of study participation were excluded from the filmed activity and participated in an alternative activity not being filmed elsewhere in the gym hall, and rejoined after recording. Pseudonyms were used to protect the identities of all participants, including the replacement of student names in interview transcripts.

## 3 Results

**Figure 2:**
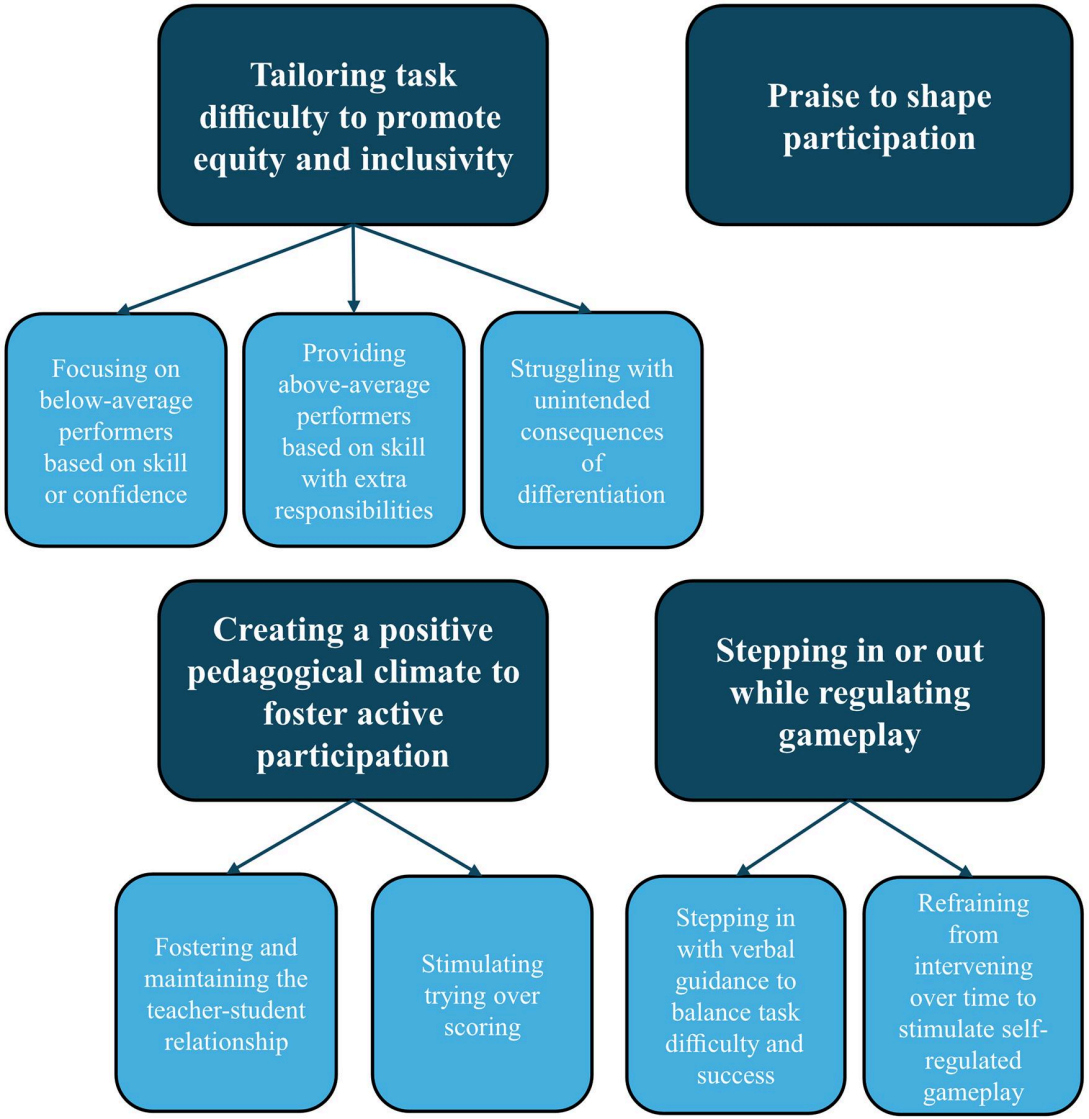
Overview of themes and subthemes.

Four themes were developed: (a) tailoring task difficulty to promote equity and inclusivity, (b) creating a positive pedagogical climate to foster active participation, (c) praise to shape participation, and (d) stepping in or out while regulating gameplay (see Figure 2). Together, these themes describe how and why PE teachers differentiate task difficulty given students’ diverse needs and skill levels in a dynamic game setting. Teachers expressed a desire to create learning environments that promoted process over result, teacher-student connection, and inclusive gameplay. They noted often intervening verbally during gameplay to support the participation of students that they perceived as less-skilled or confident, with the aim of creating success experiences for all students. While teachers reported frequent intervention, they also expressed a desire to foster more self-regulated play by minimizing interference to preserve learning opportunities and enjoyment during gameplay.

### 3.1 Tailoring task difficulty to promote equity and inclusivity

Teachers described PE as an environment in which every student should have the opportunity to participate. They expressed a desire to ensure that no student should face a task difficulty that was too high relative to their skill level. To achieve this, they emphasized the importance of differentiation in guidance, especially by providing instruction and praise according to students’ individual characteristics and skill levels. Teachers reported paying particular attention to students they perceived as performing below average in terms of skill or confidence (hereafter referred to as below-average performers). Teachers explained doing this to ensure that below-average performers could take an active role in gameplay and were not overshadowed by peers who had already mastered the game's tactics and techniques (hereafter referred to as above-average performers). It should be noted that these terms reflect teachers’ own perceptions and categorizations of their students. Teachers described praising below-average performers for actions that would not typically be highlighted for above-average performers, while also adapting game rules to limit the latter from monopolising play. Teachers described such strategies as creating the preconditions for inclusive gameplay. As one teacher explained:

Teacher 3: I'm not always objective and fair. This is an example of what I always try to do: how can I help someone who is smaller or weaker to have an equal playing field within the physical education class?

Simultaneously, teachers recognized that adaptations of task difficulty could highlight skill differences between students. Three subthemes further describe how teachers approached this tension: (a) *focusing on below-average performers based on skill or confidence*, (b) *providing above-average performers based on skill with extra responsibilities*, and (c) *struggling with unintended consequences of differentiation*.

#### 3.1.1 Focusing on below-average performers based on skill or confidence

Teachers noted feeling responsible for ensuring that below-average performers were involved in gameplay. They contrasted the goals of PE with those of competitive sport, emphasizing collaboration in mixed-skill teams over individual performance:

Teacher 7: If you try to pass the ball to someone who isn't very good at soccer and that person loses it, I'll give you a compliment for passing it to someone who finds it difficult. You could have dribbled yourself or passed it back to your friend who also plays soccer, but I don't really want that in class. You can do that on the soccer field tonight during practice or on the weekend.

Teachers described making gameplay more accessible for below-average performers by, for example, providing one-on-one instructions during gameplay:

Teacher 8: I said: “Stay there” [on offense], because M has trouble finding space, and then I think: “Yes, M, the best thing you can do is move forward”. … Many children who are not good at a sport often move to the back [of the playing field], are put in goal, or play defence.

The term ‘below-average performers based on skill or confidence’ indicates that teachers attended not only to students with lower technical or tactical skills, but also to less assertive students who may withdraw quickly when receiving feedback. Teachers noted that these students could perceive feedback as criticism. Therefore, teachers described ’sandwiching’ feedback between compliments, explaining this as a way to maintain engagement while still offering corrective input. Teachers explained this approach as allowing them to maintain engagement while still offering feedback:

Teacher 7: E has already caught the ball twice, but instead of throwing it to someone else, she threw a high ball over the defender, and both times, I deliberately didn't say anything. She's a girl, and if you say something to her, she tends to retreat into her shell and think, “I'm not going to do anything anymore, because then he (the teacher) will say something to me again”. So I try to give her three compliments first, and once I've given her three compliments, I see if I can give her some feedback on what she needs to do.

Teachers described girls as more easily overlooked or less assertive during gameplay, particularly in mixed-gender teams. They described intervening to make these students more visible, ensuring they received the ball and had opportunities for success:

Teacher 9: E is a very timid, quiet girl. … She's not a standard game player, but she always sees where she is free to receive the ball. R sometimes thinks, “I only play with the boys”, and ignores her, which is a shame. So you have to make him aware that she's standing there … which would have resulted in a fantastic combination. I want to make her a bit stronger so that she might start to make herself be heard more in the game.

Teachers’ descriptions of ‘below-average performers based on skill or confidence’ therefore encompassed not only students with lower technical or tactical skills, but also those needing additional encouragement during gameplay.

#### 3.1.2 Providing above-average performers based on skill with extra responsibilities

Teachers also described giving above-average performers additional responsibilities to promote inclusivity. For instance:

Teacher 6: I'm adjusting the assignment for him [the student is only allowed to score once one of their teammates has scored first], because his teammates can't get into scoring position because he's constantly occupying that scoring position, which also prevents them from taking up free spaces. So, I want to make it possible for them to score too. I still want to give him [the student who is constantly occupying the scoring position] the opportunity to score, and I also hope that he will now see and help his teammates to score. If he succeeds, he can score again.

In this example, the teacher introduced a new game rule, limiting how often one student could score. Beyond this rule, the teacher encouraged the above-average performer to support his teammates, implicitly taking on a coaching role. While aiming to challenge above-average performers, teachers described simultaneously attending to below-average performers, using peer coaching with the intention of enhancing their gameplay and confidence.

When teachers challenged above-average performers, they mentioned simultaneously attending to the needs of below-average performers within the same game sequence, making sure that they could keep up, as illustrated below:

Teacher 14: “Who's going to catch it?” M is standing in the far-right corner at an impossible angle to throw the ball into the tchouk, but as a handball player, I thought …, maybe with some kind of jump shot [she might be able to throw it in]. But, [at the same time], also mentioning: “Who's ready to catch it”?

Teachers described differentiation as a way to enable collective success and enjoyment in mixed-skill teams rather than as a means of separating learners.

#### 3.1.3 Struggling with unintended consequences of differentiation

Despite teachers’ best intentions, they acknowledged that differentiation may actually be the opposite of how students wish to be treated:

Teacher 7: … a lot of students don't actually want personalization, because tailored means you're different, that you're special, on whichever side of the spectrum …. Actually, everyone just wants to be the same as everyone else, playing by the same rules as the rest.

Teachers recognized that grouping or adjusting rules based on skill level could unintentionally highlight differences, prompting comparisons between students and thereby reducing their sense of competence:

Teacher 3: So, by asking these questions, I try to get the children to say: “But what do you actually see?” Of course, they are constantly aware of what the others are doing, so they say: “I'm not good enough”.

Teachers described trying to balance between addressing students’ diverse skill levels and fostering their confidence. They noted sometimes forming homogeneous skill groups to tailor the task difficulty, while remaining cautious not to overemphasize skill differences.

Another concern for teachers was whether the differentiation they used for above-average performers, for example, assigning them a coaching role, truly challenged them. Teachers questioned whether this approach primarily benefited below-average students instead:

Teacher 3: I am well aware that some of the children who are good at it will never have a worthy opponent in a classmate because they are too skilled. I say this while at the same time I am trying to protect them [lower-skilled students]. … for their enjoyment during the game, I should not let them play too much with the other children who are at a higher level, since, in my opinion, this would be undesired for their success. I would also choose the less-skilled students more often than the higher-skilled ones; that is ingrained in my system.

Teachers’ descriptions suggest that, in practice, differentiation did not always challenge all learners.

### 3.2 Creating a positive pedagogical climate to foster active participation

In line with teachers’ focus on creating an inclusive classroom environment, teachers emphasized that achieving engagement also required fostering a climate characterized by mutual respect and a sense of emotional safety that enabled all students to engage:

Teacher 4: Because I think it's important that there is always a good working atmosphere.

What teachers described as a good ‘working atmosphere’ was outlined through two subthemes: (a) *fostering and maintaining the teacher-student relationship*, and (b) *stimulating trying over scoring.*

#### 3.2.1 Fostering and maintaining the teacher-student relationship

Teachers explained that verbal guidance during gameplay was not only used to clarify expectations, guide actions, or increase confidence, but also to signal presence and connection:

Teacher 7: For me, my goal for every lesson is to briefly speak with every student in the class, give them feedback or a compliment or whatever, so that I have really seen every student.

Although teachers did not explicitly identify this as their goal when asked about their lesson goal, many described an intention to ensure that every student felt noticed. Teachers viewed connection with students as essential because of the reciprocal nature of the teacher-student relationship, meaning that to elicit effort from students, teachers felt they must also invest attention and care:

Teacher 12: In any case, I personally enjoy interacting with students, and I think it's also important for the student to have a good relationship with you. Not only because of the relationship itself, but also because many children do things for you. … If you want them to do something, they'll do it for you.

Teachers expressed this relationship as requiring not only encouragement of students’ engagement, but also genuine interest in them as individuals. Teachers described how showing appreciation and care could enhance students’ enjoyment:

Teacher 2: Reignite their enthusiasm and let them know that you are there for them and that you care about them and their actions.

Teachers believed that motivation and confidence to participate increased when their students felt seen and valued. Teachers also emphasized the importance of non-verbal presence. Physical proximity or simple gestures, such as a thumbs-up, were seen as ways to show attentiveness and encouragement. When this was not possible, such as when students were physically distant, teachers described trying to compensate by offering verbal recognition across the PE hall:

Teacher 11: If possible, I deliberately try to give a compliment across the whole hall from time to time, so that they don't feel that I'm only watching them when I'm nearby, but also when I'm a little further away.

Teachers described such verbal and non-verbal actions as ways to maintain connection and trust, which they considered important for student engagement.

#### 3.2.2 Stimulating trying over scoring

Alongside fostering teacher-student relationships, teachers described the importance of creating an environment in which students felt safe to make mistakes:

Teacher 9: And I hope they notice that I don't get angry when they don't succeed, but that they will see that he [the teacher] finds it annoying when they don't try. I still don't really get angry, but I do want them to realize that nothing is wrong, as long as you try.

Teachers viewed mistakes as learning opportunities rather than failures. They sought to normalize errors and praise students’ effort, thereby protecting confidence and motivation to keep trying. Teachers described this as a way to maintain students’ willingness to engage despite making mistakes.

Teachers also noted trying to manage overly competitive attitudes among students, recognizing that an excessive focus on scoring or winning could create an emotionally unsafe environment where students feared negative reactions from peers. This was evident when teachers observed that some students prioritised individual success over collective play. As illustrated by the following teacher:

Teacher 6: His reason for dribbling is that he is very focused on scoring, while in this case, L was definitely free to receive the ball. So, he doesn't always make the choice that is in the best interests of the team.

In response to such behaviours, teachers described the wish to promote a climate in which success was defined by engagement and effort rather than performance outcomes. Teachers mentioned reinforcing enjoyment, trust, and emotional safety as ways to sustain motivation and help ensure that every student felt comfortable engaging in gameplay.

### 3.3 Praise to shape participation

Praise had already appeared in previous themes as a pedagogical tool to enhance students’ confidence, support teacher-student relationships, and motivate students after mistakes, thereby fostering a climate centred on enjoyment. Its frequent and consistent use across all themes, combined with the variety of purposes teachers described for its use, warranted dedicated attention as a distinct theme. Teachers described praise as serving multiple purposes simultaneously: recognizing performance, shaping participation, supporting students who felt overlooked, and reinforcing desired behaviours. One teacher explained why praise was so deeply embedded in their teaching:

Teacher 12: Every child likes to hear when they've scored a goal, made a nice pass, or just played well. Every person, every child likes to hear that from someone. In this case, I'm their teacher, so I'm the one who does that. That's just because people like to hear that.

Besides recognizing correct technical or tactical performance, teachers described praise as a multifaceted tool. It served not only to confirm students’ successful actions but also to shape and sustain their engagement throughout gameplay.

Teachers noted using praise strategically to guide and reinforce desired forms of participation. One approach involved positioning students as role models through praise:

Teacher 9: He's already automatically doing well, but I also hope to achieve that this will make others see: “Hey, so that’s what he did, and it was good, I can try that too”.

Here, teachers used praise to prompt imitation among peers by highlighting one student's successful behaviour. Teachers described using praise in this way with the expectation that peers observing the compliment might be motivated to replicate the highlighted behaviour.

Praise was considered by the teachers as particularly important when students’ efforts went unnoticed by peers. Teachers noted that below-average performers, especially in mixed-gender or teams partly based on friendships, were overlooked during gameplay. Teachers described using praise as a form of recognition in situations where peer validation was absent:

Teacher 6: After the lesson, I'll go up to her and ask, “What were you doing there? That wasn't normal, was it?” Then you see those eyes twinkling with pride, as if to say, “I don't really know how I did it myself, but I did it.”…. And it's also nice because she doesn't receive compliments very often, not even from her classmates, because she doesn't excel in terms of skills, and other classmates are more likely to be chosen, so this is really a moment to put her in the spotlight.

Teachers described observing that students preferred to play with friends, and boys tended to pass primarily to other boys. As a result, some students who positioned themselves in a free spot did not receive the ball, and thus went unacknowledged by their peers. Therefore, teachers associated praise with students continuing to engage in effective gameplay behaviours rather than showing signs of demotivation or disengagement.

Teachers believed that timely praise was crucial for helping students understand which specific actions were being reinforced:

Teacher 4: She grabs the ball and starts running. It's great that L is doing that, so I have to jump on it right away and compliment her. … To stimulate them to do more of that.

Teachers believed that praise had the greatest impact when the reason for it was still fresh in students’ minds. One teacher humorously compared this process to a ‘Pavlov reaction’:

Teacher 9: That he will display this behaviour more often. So yes, the ringing and drooling, to put it bluntly, [a Pavlovian response].

Additionally, teachers described using praise to build students’ internal sense of competence and confidence over a longer time frame:

Teacher 4: She finds it very difficult to run with the ball, but she runs a little, and that gives her a small opportunity to pass the ball, because she also creates space away from the opponent. This allows her to pass the ball well. So, I think it's important to tell L: “I told you” … “You can do it.”

Overall, teachers noted using praise to position students as role models, to immediately reinforce desirable gameplay behaviours, and to build their confidence over time.

### 3.4 Stepping in or out while regulating gameplay

Across all previously discussed themes, teachers described continuously balancing when to step in with guidance and when to refrain from intervening to let self-regulated gameplay unfold. They aimed to sustain engagement, build and maintain teacher-student relationships, supporting different skill levels, and simultaneously preserve the inherent enjoyment coming from gameplay itself. One teacher summarized this dynamic:

Teacher 14: You just guide the game with your voice. Initially, it’s mainly to get the game going, so that everyone knows a little bit about what they have to do, and then hopefully you can gradually withdraw from the game, and they will start to figure it out themselves.

Teachers described establishing clear rules and structure as important for ensuring gameplay could flow. They explained, beginning with simplified versions of games and aiming to reduce their verbal presence as the lesson progressed. Some teachers noted, however, that this was not always possible. The process by which teachers regulated the degree of intervention during the lesson was further elaborated through two subthemes: (a) *stepping in with verbal guidance to balance task difficulty and success*, and (b) *refraining from intervening over time to stimulate self-regulated gameplay.*

#### 3.4.1 Stepping in with verbal guidance to balance task difficulty and success

Teachers described often intervening during gameplay by providing verbal feedback. When they sensed that verbal feedback given during gameplay was not being taken up, they froze gameplay to explain or demonstrate a situation in real time. Teachers noted that these intervention moments were aimed at simplifying a situation, guiding students toward what was expected, or clarifying tactical or technical understanding:

Teacher 5: What I'm going to do here is to put them [in the game situation] to simplify the situation, … so they know how to play. Of course, I could also explain: these are the lines, but that would just be a mess in their heads, so that's why I'm making it simpler.

Teachers noted that guidance methods were adapted to students’ learning preferences. Some required a visual demonstration, for others, verbal guidance was sufficient. Teachers explained combining verbal and visual guidance with the aim of ensuring all students understood the explanation given. Teachers reported using this approach more often in pre-vocational classes than in senior secondary school and pre-university education classes. In the latter, teachers noted often employing a questioning approach to prompt students’ reflection and understanding:

Teacher 13: Well, I'll just repeat: “What was your task? F, what should you have done?” I try to do that in a questioning way, so I try to ask a question, “What was your task?”, so that students get the idea, “Oh yes, I have to do that,” so that they hopefully apply that to the game right away and understand that “my buddy has the ball, no one is defending him, he can score just like that.”.

With this questioning approach, teachers described the wish to blend guidance while activating students’ evaluative thinking.

Especially at the start of gameplay, teachers noted feeling the need to provide close guidance to ensure that students understood the rules and structure of the activity:

Teacher 13: I also think it's important that they understand what they have to do, because this is their first korfball lesson. … That's why I'm keeping a close eye on them at the beginning.

Time management during explanations was a key concern for teachers. Some teachers explained deliberately phasing their instruction to avoid cognitive overload. An example is where they provided only the essential rules at the start and added further explanations during the game itself:

Teacher 7: You can try to go over all the rules with them at the beginning of such a game, but then they will have forgotten half of them, and some of these things will come up naturally during the game, and then you can help them.

#### 3.4.2 Refraining from intervening over time to stimulate self-regulated gameplay

As highlighted in the opening quote of this theme, teachers described typically aiming to give more verbal guidance at the start of a lesson, and then gradually wished to reduce their involvement when gameplay was flowing. For instance, one teacher noted deliberately withholding the explanation of a tactical element:

Teacher 7: I deliberately haven't said anything yet about where you should stand on the field, because you'll notice that they'll run into each other, which isn't a problem at all in the beginning.

Teachers gave two main reasons for refraining from intervening. First, they believed that excessive interference could disrupt students’ intrinsic motivation to stay engaged:

Teacher 3: But this is also why I don't always intervene, because those children are eager to participate. … They want to play and don't want the teacher to interrupt the game every time.

Teachers observed that students’ inherent enthusiasm for playing fostered a positive cycle of engagement. They therefore sought to protect this momentum by limiting unnecessary interruptions once gameplay was running smoothly.

Second, teachers believed that maintaining enjoyment supported student motivation to remain engaged, and that this, in turn, created more opportunities for discovery and learning through gameplay. As one teacher explained:

Teacher 4: You shouldn't prescribe everything down to the last detail. Discovering things for yourself is also a very good way of learning.

Teachers expressed a hope that encouraging self-regulated and enjoyable gameplay within lessons would also inspire students to remain active outside of school:

Teacher 14: I like it when they're at the campsite and they can play independently.

## 4 Discussion

This qualitative study addressed the research question: *How and why do teachers adapt task difficulty during junior secondary school game-based physical education lessons?* Teachers primarily discussed differentiating task difficulty through verbal guidance. They supported 12–14-year-old students they perceived as below-average in meeting tactical or technical demands, based on their ongoing evaluations of students’ confidence and engagement. Interventions aimed to build positive relationships, normalize mistakes, and emphasize effort over scoring, fostering inclusive and motivating gameplay.

Before interpreting our findings, we acknowledge the study's strengths and limitations. A key strength is the use of stimulated recall interviews to access teachers’ in-the-moment decision-making. This method allowed teachers to reflect on specific teaching moments and explain their reasoning when differentiating task difficulty. Also, the study focused on game-based lessons, which make up the largest part of PE curricula worldwide (5). On the other hand, the study comes with limitations that impact the generalizability of our findings. First, the teacher sample was drawn from a single national context, teaching students in junior secondary school, which limits the generalization of findings to other educational contexts. Second, our sample and the selected lesson might be biased. Teachers who chose to participate may be more reflective about their teaching, which could limit the generalizability of findings to other PE teachers in the Netherlands. Lastly, as a single researcher embedded in the same Dutch educational context as the participating teachers, certain assumptions, such as the importance of enjoyment and confidence as prerequisites for learning, may have felt familiar. A researcher with a different cultural identity might have interpreted the same data differently.

While teaching games for understanding interventions are mostly designed around manipulating task constraints, such as adding an extra player, playing with a softer ball, or applying different scoring roles (13–16) teachers during the interviews mainly discussed using verbal guidance to differentiate task difficulty, despite the interview protocol including examples that prompted reflection on a wider range of adaptations. Their main strategies included one-on-one directive feedback, demonstrations, and reflective questioning used to simplify tactical or technical elements of gameplay. While questioning was present, teachers reported that time constraints made it difficult to sustain, often leading them to ’spoil’ the answer to resume gameplay quickly. So, although teaching games for understanding provides a wide repertoire of task constraint manipulations, teachers tended to differentiate task difficulty through more immediate verbal guidance during games. This highlights a tension between the hands-off approach promoted by teaching games for understanding and teachers’ practical concerns about efficiency, lesson flow, and positive teacher–student relationships.

Moreover, throughout the interviews, praise emerged as more than merely a pedagogical tool to influence students’ enjoyment and motivation. Although praise appeared across all four themes, its analytical significance extends beyond frequency. In this sense, praise operated as a verbal strategy through which teachers lowered task difficulty, describing praise as the best ‘tip’ to learn from. This role aligns with operant conditioning, where consistent and timely feedback strengthens the likelihood of desired behaviors recurring (29). Praise was additionally used to portray skilled behavior as role model behavior for other students to copy. This reflects the idea of observational learning and aligns with Bandura's social learning theory, which describes how role models could transmit knowledge and teach peers effective skills and strategies for managing environmental demands (30). Therefore, due to the inductive nature of the analysis process, we identified praise as a multifaceted and deeply embedded tool in the teacher's toolbox for lowering task difficulty.

The reliance on verbal guidance and the widespread use of praise suggest that teachers in this study functioned predominantly as what we term motivational coaches. This role is characterized by praise and individual verbal guidance aimed at motivating and directly instructing students, rather than the design of task constraints and guided discovery envisioned within teaching games for understanding, where verbal guidance is meant to prompt students’ tactical awareness without prescribing solutions. This emphasis on verbal coaching may reflect a prioritization of the motivation and confidence dimensions of physical literacy (2), as suggested by the themes, tailoring task difficulty to promote equity and inclusion, creating a positive pedagogical climate to foster active participation, and praise to shape participation. While task constraint manipulation and verbal feedback can both be tailored to individual students, verbal feedback is quicker to deliver and less disruptive to the flow of the game, making it a more time-efficient tool for individually targeted motivational support. This flexibility may explain why teachers gravitated toward verbal guidance, as their reasoning reflected a prioritization of equity and inclusion that required attending to individual students’ motivation and confidence. However, this is in contrast to teachers verbally facilitating discovery as envisioned in teaching games for understanding. This tension was evident in the stepping in or out while regulating gameplay theme, in which teachers described conflict between intervening verbally to support individual students’ participation and refraining from intervention to preserve the enjoyment gameplay gives for the group and self-regulated gameplay. The role of motivational coach, thus, is in tension with teachers’ training as skill designers who manipulate task constraints and facilitate verbally guided discovery to promote the development of physical competence.

We consider two factors that may underlie why teachers adopted the role of motivational coach: the developmental stage of their students and the use of psychological theory in educational practice. Our results stand in contrast to earlier research in which elementary school PE teachers with five or more years of experience oriented their interactive decisions primarily toward skill acquisition (22). The 12–14-year-old students in our study may have presented a greater motivational challenge than the younger children in the study by Housner and Griffey. Adolescence is characterized by heightened sensitivity to peer evaluation and increased salience of social identity, rendering 12–14-year-olds more susceptible to disengagement when they feel overlooked, embarrassed, or outperformed by peers (31). This vulnerability is particularly concerning given that physical activity levels decline sharply during adolescence, with over 80% of adolescents globally failing to meet recommended daily physical activity guidelines (32, 33). The risk of disengagement and dropout from physical activity during this developmental period may therefore place additional pressure on PE teachers to prioritize motivational support, potentially at the expense of the skill-oriented discovery-based approach envisioned within teaching games for understanding. This was reflected in teachers’ descriptions of focusing on what they perceived as below-average performers who were at risk of being overlooked during gameplay. It is worth noting, however, that in the study by Housner and Griffey (22), teachers were explicitly tasked with improving students’ skills in soccer and basketball dribbling. This context may have foregrounded skill acquisition in ways that everyday game-based practice does not. Besides the developmental stage of the students, the use of psychological theory in education might have pushed teachers into the role of motivational coach. Psychological theory, such as the self-determination theory, is developed to describe an individual student's needs for competence, relatedness, and autonomy to foster motivation (12), needs clearly reflected in teachers’ descriptions of praise, teacher-student relationship, and emotional safety. However, the self-determination theory's focus on individual psychological needs may leave teachers without a clear framework for managing motivation at the group level, where, during game-based lessons, the teacher needs to motivate the whole group.

It is important to note that the first author's positionality may have shaped the interpretation that teachers, in their reasoning, prioritized motivation and confidence. Having followed PE lessons within the Dutch PE curriculum and identified as higher-skilled myself, I may have been more attuned to teachers’ prioritization of below-average performers, and therefore more inclined to foreground this pattern in the findings. However, teachers distinguishing between students based on skill level aligns with observations by Dutch PE researchers (6). They found that teachers implicitly categorize students into distinct groups based on their judgments of skill level, gender, and ethnicity, often without realizing the assumptions underlying those categorizations. This pattern also resonates with qualitative findings from other Western contexts: Lewis (20) reported that English PE teachers prioritized relationship-building and appropriately matched challenges to students’ skill levels and preferences to motivate participation, while Larsson and Nyberg (19) found that Swedish teachers emphasized enjoyment over technical improvement and felt hesitant to push students too far for fear of undermining motivation. This cross-national pattern may reflect a broader cultural specificity: the prioritization of enjoyment as a foundation for learning may be characteristic of Western, individualistic, economically wealthy contexts in which attainment of personal goals is particularly valued (34).

The observation that Dutch teachers in their reasoning considered feelings of competence as a motivational precondition for learning is theoretically interesting. Vygotsky highlights that task difficulty should be set slightly above a student's current skill level to promote learning (11). Self-determination theory posits that motivation results from fulfillment of a psychological need for competence, understood as a felt sense of confidence and effectance in action that leads people to seek challenges optimal for their capacities (12, 35). A simplistic interpretation of the two theories puts the teacher in a dilemma: if perceived competence is a motivational precondition to develop physical competence, how can students be sufficiently challenged to become competent? However, these two frameworks are less contradictory than they first appear. Vygotsky's zone of proximal development does not simply recommend challenge; it recommends challenge combined with guided support (11), and it is precisely this support that can help students experience success within difficult tasks, thereby building their perceived competence. Similarly, according to self-determination theory, students do not need to feel fully capable for their need for competence to be fulfilled (35). In fact, students feel more competent when succeeding at a task that is perceived as difficult compared to when succeeding at a task that is perceived as easy (36). The tension, therefore, is not fundamentally between the two frameworks, but in how teachers translate both into practice. The teachers in our study resolved this tension by prioritizing perceived competence through reduced task difficulty rather than by supporting students through challenging tasks. Their reasoning is understandable: Ryan and Deci (35) describe that positive feedback can enhance motivation via perceived competence. However, this effect operates in the context of appropriate challenge, not reduced challenge. Lowering task difficulty to protect perceived competence is therefore not the only nor necessarily the most effective response available to teachers. An alternative approach would be to set tasks within a student's zone of proximal development while simultaneously supporting perceived competence by acknowledging effort and progress, and by buffering the inevitable mistakes that accompany challenge rather than avoiding challenge. This would allow physical competence to develop at the same time.

Interestingly, teachers struggled to answer the final interview question: ‘How challenging do you think this lesson was for your students?’. This struggle suggests that, although teachers can adapt task difficulty by adjusting task constraints in the learning environment, challenge itself is not an inherent property of a task. Rather, it is a subjective experience shaped by students’ interpretations of that difficulty (37). Qualitative studies offer insight into students’ experiences of challenge, and indicate that students feel insufficiently challenged during PE (37–39). For instance, children around ages 11–14 start to see ‘fun’ differently: rather than simply enjoying games, they start to value being challenged, improving skills, and learning something new (38). Walls (37) further showed that students reported repetitive activities becoming insufficiently challenging once a certain level of mastery had been reached. Rikard and Banville (39) reported comparable results, finding that students felt more challenged when engaging in unfamiliar activities than in familiar ones. When difficulty is primarily adjusted to protect confidence and ensure inclusive participation, it becomes difficult to determine whether all students encounter sufficient and meaningful challenge. Teachers’ struggles to answer this question underline the need for student perspectives to be incorporated into future research.

## 5 Conclusion

This study suggests that secondary school Dutch PE teachers, when reasoning about differentiating task difficulty in game-based lessons, functioned less as skill designers – prioritizing tactical and technical development, and more as what we describe as motivational coaches, whose rationalization of moment-to-moment decisions placed students’ confidence and motivation at the forefront. Compared to the physical competence, knowledge, and understanding domains of physical literacy, teachers placed more emphasis on confidence and motivation, from which they believe learning may flow. These findings extend the literature on PE teacher interactive decision-making in naturalistic secondary school game-based lessons. Moreover, our findings suggest that teachers tended to set lower task difficulty to protect all students’ perceived competence and motivation rather than stimulating all students equally. Drawing on self-determination theory and Vygotsky, we propose that acknowledging students’ effort and progress may help maintain perceived competence while still providing challenge, supporting the simultaneous development of motivation and physical competence.

Practically, these findings suggest that Dutch PE teacher education may need to move beyond introducing teaching games for understanding as a conceptual toolkit toward developing teachers’ capacity to use its full range of tools, including a closer attention to whether group motivation is best maximized by attending to individual motivational needs. Future research should complement teachers’ perceptions of differentiating task difficulty with adolescent students’ perspectives on how this shapes their experienced challenge, motivation, and learning.

## Data Availability

The datasets presented in this article are not readily available because of privacy considerations. Participants provided consent for their interview transcripts to be shared upon reasonable request with researchers conducting comparable research. Requests to access the datasets should be directed to Joyce E. Burger, j.e.burger@vu.nl.

## Supplementary material

Supplementary File 1 Interview protocol.

Supplementary File 2 Researcher interview manual.
