# Supplementary material for: Orchestrating gameplay in Dutch physical education: how and why teachers regulate task difficulty
Source: Front Sports Act Living. 2026 May 29;8:1837783. doi: 10.3389/fspor.2026.1837783 (PMC13260430; doi:10.3389/fspor.2026.1837783)
Supplement: Supplementary File 1 — Interview protocol. [file Supplementaryfile1.docx]

# Interview protocol

**Design of the lesson**

WHAT

Why this exercise?

HOW

Why this organisation?

What was your lesson goal?

**2 components of the answer**

**Elements making the task more difficult or easier**

Why did you perform that action?

What was your action?

?

Changing arrangement

Changing of exercise assignment

Verbal guidance

**After the lesson**

How challenging do you think this lesson was for the students?

Was this a representative lesson?
