# Supplementary material for: Orchestrating gameplay in Dutch physical education: how and why teachers regulate task difficulty
Source: Front Sports Act Living. 2026 May 29;8:1837783. doi: 10.3389/fspor.2026.1837783 (PMC13260430; doi:10.3389/fspor.2026.1837783)
Supplement: Supplementary File 2 — Researcher interview manual. [file Supplementaryfile2.docx]

# Protocol surrounding the interview

This manual is intended to record the tasks of the researcher surrounding each interview in order to structure this process.

**Items needed:**

• Handycam camera, including USB connection cable and charger.

• Handycam tripod.

• GoPro camera.

• Headset including screw for the GoPro.

• USB stick for GoPro SD card.

• USB cable to connect the GoPro to the computer.

• USB cable to connect phone to laptop.

• Mobile phone.

• Laptop.

• Laptop charger.

• Mobile phone charger.

• Interview protocol.

• Notebook for notes.

• Pen.

• This protocol.

## Preparations before the interview

It is important to schedule a brief phone call with the Physical Education (PE) teacher to discuss the final details. These details are as follows:

- What time does the lesson start? And what time should I be there?
- Location of the school – exact address.
- Which game activity has been chosen.
- Procedure (two questionnaires – demographic details and informed consent) and setting up the GoPro camera on the head, and making a test recording.
- Ask if any students are not allowed to be on the video recording and, if necessary, immediately discuss the solution for these students.
- Ask for the Wi-Fi code so that the Quick app can be connected to the GoPro camera.
- Finally, it is also useful to find out in advance when the chosen activity will take place. It is also useful to ask how the PE teacher will indicate when the recording can start. It is a good idea to confirm this on the day itself.
- Check all equipment and charge it properly.

### Checking the equipment and measurement data

- Fully charge the camera and GoPro camera (the red light on the GoPro camera should no longer be lit).
- Film a clip of approximately 20 minutes with both cameras (can be shorter with the backup camera).
- Also, make sure you check the GoPro quick app for connection and whether it works.
- Upload both files to your laptop and to the research drive to check whether this works.
- Also, try to record audio throughout the recording with both your laptop and mobile phone to check that the sound is coming through properly.
- Check that all Qualtrics links are working and can be filled in beforehand.
- Go through the protocol beforehand and bring a printed copy with you --> including a laminated interview protocol.

## On the day itself

### Before the start of the lesson

- Fill out the informed consent form.
- Fill out the demographic details questionnaire.
- Have the GoPro camera placed on the teacher’s head and adjust it so that it fits comfortably.
- Make a test recording via the Quick app to check whether it is recording.

### During the lesson and filming

- During the PE lesson, try to stay in the corner near the backup camera. That way, you will interfere as little as possible with the PE lesson itself, and it will remain as natural as possible.
- At the same time, try to keep an eye on what is happening during the lesson and make a note of anything that stands out so that you can keep a log. Try to write these notes down in a notebook that you keep with you. Later, you will transfer these notes to the memo within the Atlas.ti environment.
- When the GoPro is turned on, it is helpful to have the teacher close by so that you can hear the beep and know that it is actually on. Do this as much as possible out of sight of the students and then return to the backup camera.
- During the video recording itself, try to keep track of interesting moments for yourself by writing them down, because closing the app can sometimes cause the connection with the GoPro to be lost. Also, try to orient yourself to moments when you would pause the recording and look for good points to do so (end of explanation or intervention) and note these in the notebook as well.

### Before the interview

- First, try to transfer the video recording to your laptop via the SD card and play it back to check the recording.
- Once you have done this, you can immediately delete the recording from the backup camera.
- Also, try to find a good spot for the interview and check whether the recording is working.
- Check whether the data has been saved in Qualtrics, because if not, you can have it filled in again if something went wrong.

#### Instructions before the interview

I will be audio recording this interview, so I will start recording now.

We will review the lesson in a moment, but before we do that, I have three questions about the choices you made prior to this lesson. I will ask you each of these questions:

The first question is: “Why did you choose this exercise?”

The second question is: “Why did you choose to organize this exercise in this way?”

The third question is: “What was your goal during this lesson?”

--------------------------------------------------------------------------------------------------------------------

Now that you have answered these questions, we will review the recorded video footage together. You will be asked to pause the recording every time you make the exercise easier or more difficult for the students. This could include moments when you changed the assignment of the exercise, gave verbal guidance, or adjusted the arrangement. Also pause at other moments when you made the lesson easier or more difficult, but when this does not fall under one of the three examples mentioned (hence the ‘?’ in the fourth round within the interview protocol). It is important to do this every time, even if a situation occurs several times during the lesson. After you have paused the video recording, I would like to hear in your answer to “what your action was” and “why you performed that action.”

In addition to you being able to pause it, I can also pause it. When I do so, I will ask you to answer the same two questions about the moment I paused it, i.e., “What was your action?” and “Why did you perform that action?”

Is this clear? Do you have any questions (otherwise, provide clarification with an example).

Okay, let's begin.

#### During the interview

Check in advance whether you can be heard clearly on the audio recording and make a test recording for this purpose. Furthermore, the table below serves as a guideline for the researcher regarding pausing the video recording based on the described moments that may occur during the PE lesson. This table is constructed based on pilot studies. These moments serve as a guideline for consistently pausing the recording at similar moments during different PE lessons. In addition, as a researcher, you should only pause at these moments if this has not been done by the PE teacher themselves.

A few general tips regarding pausing the video recording:

- Try to pause the video recording during each interaction between the teacher and student in which the teacher addresses something related to the lesson content (clarifying rules, for example). When several students ask questions, try to cluster them and pause the video after this interaction is over.
- Try to pause at the end of each such moment as much as possible. If you accidentally pause a little later and the teacher says something else captured on video that is not relevant, this is not a problem.
- When the teacher looks ahead to what is coming later in the video recording to support their answer to the question, you do not need to pause the video again at that point. This is to prevent the flow of viewing from being interrupted, which can have an unpleasant effect and lead to a fragmented way of watching the video recording.

| **Action** | **Explanation** | **Example(s)** |
| --- | --- | --- |
| Verbal guidance | The teacher responds to a student’s comment or question about the game activity or asks questions to assess the student’s understanding. | Includes clarifying game rules, questioning students about technical or tactical elements of gameplay, or addressing students’ comments that indicate that something is not working. These explanations can be given to a group or an individual student. Off-task remarks such as “I don’t feel like it” or “I need to drink water” are not included. |
|  | The teacher makes comments during a game situation, often relating to events on the playing field. | Comments may be positive, highlighting something that is going or has gone well, or serve as feedback. |
|  | The teacher gives a technical or tactical tip or compliment to a team or individual student. |  |
|  | The teacher positions students within the game situation. | Directs students to the correct position on the field and may advise them on what to do and what role they can play from that position. |
| Visual guidance | The teacher visually demonstrates part of the activity. | The teacher may act as a member of a team to model a specific strategy, technique, or gameplay role, applying theory directly in practice. |
| Adjusting the arrangement | The teacher changes the team composition or the matchup between teams. | May involve moving certain students to a different team, changing who plays against whom, or assigning a different role to a student within their current team. |
|  | The teacher changes the materials used in the lesson. | May involve replacing or modifying sticks, balls, mats, or the size of the playing field. |
| Adjusting the assignment | The teacher modifies the rules of the game. | Rules may be adjusted in various ways, for example: allowing the ball to bounce once before being returned, or permitting a player to catch the ball before passing it on. |
|  | The teacher changes the objective of the game. | For example, shifting the goal from winning or achieving the highest score to keeping the ball in play as long as possible, creating a rally. Or ensuring that every student has an active role, thereby promoting cooperation. |

#### Instructions after having reviewed the video footage

That's it. I have two more questions for you to answer, unrelated to this video recording.

The first question is: “Was this lesson you taught representative of how you normally teach your PE lessons?”

The second question is: “How challenging was this lesson for the students, and please explain.”

That's it. Thank you very much for participating. I'll stop the audio recording now.

### After the interview

If necessary, complete the demographic questionnaire if there was no time for it before the measurement!

Email the debriefing to the PE teacher.

Upload the following to the research drive:

- Video recording of the lesson from the GoPro.
- Audio recording from the mobile phone.
- Audio recording from the laptop.
- Informed consent form from Qualtrics.
- Demographics .csv file from Qualtrics.
- Memo update as an Atlas.ti file.

If there is no Wi-Fi, place the above-mentioned files in the encrypted vault.

Remove the recording from the SD card of the GoPro and check it on the backup camera.

After uploading everything to the research drive, make sure you verify again that all files have been deleted from the equipment and laptop:

- Video recording on a GoPro SD card.
- Video recording backup camera SD card.
- Video recording GoPro laptop (in vault).
- Video recording backup camera (in vault --> probably not saved anyway if the GoPro camera recording was sufficient).
- Audio recording on the laptop itself (and in the vault).
- Audio recording on the phone.
- Audio recording phone in the vault (you can keep this after transcription is complete).
- Atlas.ti file downloaded for memo.
- Qualtrics informed consent and demographics questionnaire.

Return the borrowed backup camera to the technical office.
